# Accuracy and bias of high-frequency ultrasonography in measuring white spot lesion depth: an in-vitro comparison with micro-CT

**DOI:** 10.1007/s00784-026-06956-y

**Published:** 2026-06-03

**Authors:** Taylor Toothman Sulkowski, Steven Makowka, Oliver D. Kripfgans, William Tanberg, Stephen Warunek, Thikriat Al-Jewair

**Affiliations:** 1 Orthodontist, private practice, Chapel Hill, NC USA; 2 Materials Testing Facility, State University of New York at Buffalo, Buffalo, NY USA; 3 Department of Radiology and Biomedical Engineering, Medical School and College of Engineering, University of Michigan, Ann Arbor, MI USA; 4 Department of Biostatistics, School of Public Health and Health Professions, State University of New York at Buffalo, Buffalo, NY USA; 5 Department of Orthodontics, School of Dental Medicine, State University of New York at Buffalo, 3435 Main Street, Buffalo, NY 14214 USA

**Keywords:** White spot lesions, Demineralization, Depth, Ultrasound, Micro-CT

## Abstract

**Objectives:**

This in vitro study aimed to evaluate the accuracy and bias of Ultrasound (US) in measuring the depth of white spot lesions (WSLs), in comparison to micro-CT (µ-CT).

**Materials and methods:**

The study included 120 bovine maxillary incisors. Artificial WSLs were created on the facial surface of each incisor. Incisors were categorized into two groups, shallow WSLs and deep WSLs and subjected to 2-day and 4-day pH cycling protocols, respectively. All samples were imaged with both µ-CT and US using second harmonic imaging at 12/24 MHz. WSL depth was measured and the average calculated. Enamel thickness was assessed on the µ-CT images using the same methodology.

**Results:**

Mean lesion depths in the shallow WSLs group were 138.3±17.8 μm (µ-CT) and 169.2 ± 37.8 μm (US), while in the deep WSLs group, depths averaged 299.9 ± 47.7 μm (µ-CT) and 309.3 ± 75.7 μm (US). The absolute mean differences between µ-CT and US were significantly different from zero (*p* < 0.001). US consistently overestimated lesion depth compared to µ-CT in both groups, with a significant difference in the shallow WSLs group (*p* < 0.001).

**Conclusions:**

US demonstrates lower accuracy than µ-CT in measuring WSL depth, consistently overestimating lesion depths, particularly in the shallow WSLs group.

**Clinical relevance:**

The ability of a diagnostic method to measure WSLs of varying depths is clinically important, as treatment efficacy depends on lesion depth and the extent of enamel demineralization. US in non-invasive and demonstrates potential for clinical use; however, further validation is required before it can be reliably applied to the clinical assessment of WSL depth. Clinicians must also consider practical factors such as equipment cost and size, the need for coupling materials, and requirements for operator training and calibration.

**Supplementary Information:**

The online version contains supplementary material available at 10.1007/s00784-026-06956-y.

## Introduction

Demineralization leading to the formation of white spot lesions (WSLs) is common during orthodontic treatment [1]. Recent systematic reviews emphasize the need for clinicians to consider WSL risk during treatment planning and appliance selection and to monitor their occurrence using effective detection tools [2]. Accurate assessments of the depth and extent of WSLs is critical in determining the most appropriate approach to restore the enamel. While 2D radiographs are commonly used to detect caries, they have limited sensitivity for identifying intact WSLs. By the time lesions are visible on bitewings, they often have already progressed into dentin, disqualifying them as WSLs [3]. Moreover, bitewings are ineffective for detecting facial-surface lesions due to the beam’s perpendicular orientation.

Ultrasonography, which utilizes ultrasound (US) waves and measures backscatter as a function of time, is a non-invasive imaging technique with potential for diagnosing WSLs [4]. An US scanner generates electrical impulses that are converted into sound waves that travel through the body [5]. These waves are reflected back by tissues with varying acoustic impedance, enabling image formation based on the difference in tissue properties [5]. Specifically, dental diagnostic US uses acoustic (i.e., mechanical) waves above a frequency of 20 kHz to detect changes in tooth structure [6]. These acoustic waves can then be used to assess the internal properties of a tooth, such as its elastic modulus, a parameter that correlates with the mineral content of the dental tissue [7].

Multiple studies have examined the use of US to detect changes in tooth mineralization [6, 8–10]. However, only a few have specifically assessed the depth of demineralization using US and, to our knowledge, none attempted to validate the technology in a clinically applicable manner and compare it to a gold standard [5, 7, 11, 12]. Kim et al. studied the efficacy of high-frequency US (HFUS) combined with b-mode imaging in assessing the depth and morphology of WSLs, using micro-computed tomography (µ-CT) as the refence standard. Their findings demonstrated that WSLs’ morphology visualized with HFUS closely resembled that observed with µ-CT. Furthermore, there was no significant difference in the mean WSL depths measured by HFUS and µ-CT (mean difference = 77.8 μm, *P* > 0.05) [11]. However, the study had a small sample size (*n* = 4) and the HFUS technique used cannot be clinically replicated. In another study by Yanikoglu et al., US demonstrated strong concordance with light microscopy measurements, yielding an intraclass correlation coefficient of 0.97 (*p* < 0.001) [12]. The study further revealed that US could distinguish lesion depths across three different International Caries Detection and Assessment (ICDAS) classifications (ICDAS I-III), with lesion depth increasing in accordance with lesion severity, suggesting that US may be capable of differentiating lesions based on depth [12]. The study however did not measure the accuracy and bias of measurements at different depths.

While the aforementioned studies have demonstrated the capability of US to detect the depth of dentin and enamel mineralization, to our knowledge, none have quantified the accuracy (absolute mean difference) or directional bias (over or under-estimation) of clinically relevant, commercially available HFUS for measuring the depth of enamel WSLs in comparison to µ-CT [5–7, 11–13]. The aim of this validation study was to assess the accuracy and bias of US compared to µ-CT in measuring WSL depth, with the goal of informing depth-based treatment decisions. We hypothesized that US is a valid approach to assess WSL depth with no significant difference between US and µ-CT in lesion depth assessments.

## Materials and methods

### Study design

This an in vitro study that used extracted bovine incisors. Request for use of animal tissues was granted by the Institutional Animal Care and Use Committee at the University at Buffalo.

### Power analysis

To detect a medium effect size (Cohen’s d = 0.5) in the mean paired difference between µ-CT and US measurements with 90% power and a significance level of α = 0.05, a minimum of 44 samples per group (2-day and 4-day) was required. The final sample size was increased to 60 per group (total = 120) to account for any potential losses during different experimental stages. Very few such losses occurred resulting in a sample size that exceeded the minimum. Given the non-destructive nature of the testing and the shared outcome measures, all samples were evaluated using both modalities.

### Sample preparation

Sixty maxillary bovine incisors were harvested. Teeth exhibiting visible cracks or defects were excluded. Following extraction, the specimens were stored in a 1% Chloramine-T solution for one week at 4^o^ C prior to sectioning. Using a precision diamond saw (Buehler Isomet 1000, Lake Bluff, IL, USA) under water irrigation, each crown was sectioned in an inciso-gingival direction to yield 120 paired samples. The crowns were fully separated from the root. Pulp tissues were removed, and the sectioned crowns were returned to 1% Chloramine-T for an additional week of storage. Afterward, all samples were dried and embedded in a radiolucent chemical cure resin (Biocryl Resin Acrylic, Great Lakes Dental Technologies, Tonawanda, NY, USA). The acrylic blocks were trimmed to approximately 13 mm in thickness, and flat surfaces were polished on the facial aspects of the specimens using an Automet 250 Pro Semi-Automatic Grinder (Buehler, Lake Bluff, IL, USA) with wet 600-grit (P1200) silicon carbide abrasive paper (CarbiMet PSA, Buehler, Lake Bluff, IL, USA). When not in use, all specimens were stored in 1% Chloramine-T solution under refrigeration, with the solution replaced every 3 months.

To standardize lesion size, a 4 × 4 mm square sticker was affixed to the polished surface of each specimen. Using a round bur, three small reference indentations were made in a triangular configuration around the future lesion site – one positioned incisal to the lesion and two gingival. An acid-resistant nail varnish was then painted to the remaining exposed tooth surface. Once fully dried, the sticker was removed to expose the designated lesion area. All study procedures were conducted by one investigator. Study procedures are illustrated in Supplementary Figs. 1 and 2.

### White spot lesion creation and pH cycling

WSLs were created using a protocol adapted from Liu et al. [14]. The specimens were randomly assigned to two groups subjected to different durations of pH cycling to produce shallow (S-WSL) and deep (D-WSL) lesions. The demineralization solution consisted of a 0.05 mol/L acetic acid preparation (2.9 mL per L of solution) mixed with 0.16 g/L calcium phosphate and deionized (DI) water and adjusted to a pH of 4.0 with either 10 N hydrochloric acid or 10 N sodium hydroxide and monitored with universal pH strips. The remineralization solution was made of 0.07 g/L calcium chloride, 0.13 g/L calcium phosphate, 11.18 g/L potassium chloride, and DI water adjusted to a pH of 7 with either 10 N hydrochloric acid or 10 N sodium hydroxide and monitored with universal pH strips. Each specimen required 50 mL of new solution at the beginning of each phase of the pH cycling; at which point solutions were checked for pH consistency with universal pH strips.

The pH cycling process was conducted in two groups based on the length of pH cycle. Samples were loaded into large plastic containers then placed on an oscillating platform in an incubator set to 37⁰ C. All specimens were cycled between a 14-hour demineralization and 10-hour remineralization period. The S-WSLs were cycled for 2 days while the D-WSL were cycled for 4 days. Between each solution, specimens were rinsed with DI water.

### Micro-CT and ultrasound scanning

All specimens were scanned with µ-CT (µCT 100 scanner, Scanco Medical AG, Bruttisellen, Switzerland) with the following settings: 90 kV, 40.

5 µA, 0.5 mm aluminum filter and 1,500 projections per 180⁰ with a 680-millisecond exposure time. Approximately four samples were loaded into each scanning tube, which was then filled with DI water. Scans were conducted over a period of two weeks. All scans were performed by one lab professional. During imaging, the beam was oriented parallel to the lesion surface. Scans were reconstructed with Scanco software (Scanco, Nokomis Florida, USA) with a final isometric voxel size of 11.4 μm.

Ultrasound scans were conducted using a clinical HFUS system (ZS3, Zonare/Mindray, San Jose, CA, USA) equipped with an L30-8 linear array transducer, measuring 18.2 mm in wide and 15 mm in height. This transducer has been verified in previous studies for intraoral use. The transducer was operated in the default settings for ‘small parts’ and the sample was positioned axially to coincide with the elevational focal point. The US system operated within a bandwidth of 8–30 MHz, as specified by the manufacturer [16]. The system gain was set to 50. The pulse length, determined from the axial point-spread function, was measured at 66 µm. All measurements were conducted at a controlled temperature of 72 °F (22°C) [16]. Imaging was performed using second harmonic mode, with a transmit frequency of 12 MHz, and a receive frequency of 24 MHz, to obtain high spatial and suppress clutter [15, 16]. The specimens were submerged in water, used as a coupling medium, at a standardized location using a computer-controlled 3D positioning system (Anaheim Automation Inc, Anaheim, CA, USA). The transducer was positioned to 8 mm from the sample surface, i.e., to the transducer’s elevational focal distance. To acquire a 3D data volume, the transducer was swept in the elevational direction of the image plane across the sample. For each specimen this sweep range extended from the incisal to the gingival edge of the lesion, including a 1 mm margin before and past the outer boundary. A constant sweep rate of 1 mm/s was set for all samples. Given the frame rate of 15 frames per second (15 Hz) of the imaging system, 15 frames per millimeter were acquired, i.e., an elevational frame spacing of ~ 66 µm was achieved. With an elevational slice thickness of 308 µm for a 50% speckle decorrelation, we allowed for conservative elevational sampling.

### Micro-CT and ultrasound analysis

**Fig. 1:**
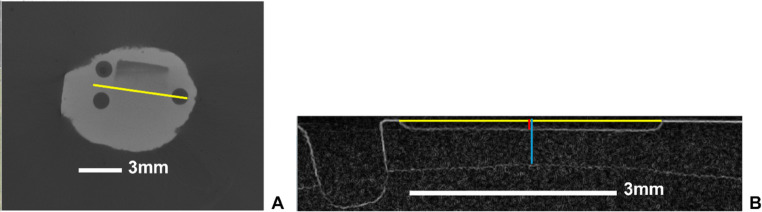
Reference line bisecting a lesion. To analyze the µ-CT images, individual DICOM files were imported into ImageJ, generating a volumetric reconstruction of the sample from a facial perspective. The examiner then scrolled through the image until the three reference points were visible and drew a line (yellow) bisecting the one point incisal to the lesion and the two points gingival to the lesion (**a**). A function called *kymograph* builder was then utilized to view the image from the sagittal position. A line (yellow) was drawn across the top of the lesion and another line (red) bisecting the first was drawn to the depth of the lesion and measured. A vertical line (shown in blue) was also drawn to measure the depth of the enamel in the same location (**b**)

1. Reproducibility of US:

A pilot study was carried out to assess the reproducibility of US measurements. Two separate US scans were obtained per specimen from a convenience sample of four specimens ranging from shallow to deep (two S-WSL and two D-WSL). Individual tag image file format (TIFF) files of the US images were loaded into ImageJ (open-source software, imagej.net) for depth measurements (Fig. 1a-b). This produced a scrollable image of the lesion in the sagittal view. From these, the facial view had to first be reconstructed. The examiner then scrolled through the image until the three reference/registration points were visible and drew a line bisecting the one point incisal to the lesion and the two points gingival to the lesion. The Kymograph Builder command in ImageJ was used to visualize the sagittal view of the images. A reference line was first drawn across the top of the lesion, followed by a perpendicular line extending from the midpoint of the first line to the lesion’s deepest point. The length of this second line represented the lesion depth. To account for variability, this measurement was repeated three times per sample by starting again at the scrollable image in the sagittal view.

2) Accuracy and Bias of US compared to µ-CT:

Once the reproducibility of US was confirmed, ImageJ was used to measure the depth of the WSLs in µ-CT and US in all 120 specimens and to also measure the thickness of the enamel in the µ-CT images. Data of three samples were eventually eliminated from the study due to corruption or loss of the US file, bringing the total number of specimens to 117 (S-WSL = 58 and D-WSL = 59) (Supplementary Fig. 1). Each measurement was obtained in triplicate and averaged for analysis. To ensure investigator blinding, all US images were measured and recorded for the entire sample, after which the measurements were concealed, by hiding the appropriate columns in the excel sheet, prior to performing the µ-CT measurements.

The analysis of the US images was the same as in the pilot study. For µ-CT analysis, the region of interest was defined using the µ-CT software, and digital imaging and communications in medicine (DICOM) files were imported into ImageJ. Enamel thickness was measured three times per sample using the same protocol. This measurement was not possible with US due to its limited resolution, which precluded accurate visualization of the enamel layer.

For qualitative evaluation, a 3D superposition of a representative sample was performed using µ-CT and US images within 3D Slicer software (open-source software, https://www.slicer.org). This allowed for visual comparison of lesion geometry across both imaging modalities. To assess the quality of demineralization, two samples from each group were randomly selected and covered with a water-insoluble mixture of 30% w/v barium sulfate and petroleum jelly and immediately imaged with µ-CT. Additionally, scanning electron microscopy (SEM) was performed using a Hitachi SU70 Field Emission SEM (Hitachi High-Tech Corporation, Tokyo, Japan) to analyze the lesion surfaces of representative WSLs. The two specimens were dehydrated in a vacuum chamber for 24 h before carbon sputter coating. Secondary electron images were obtained for one S-WSL and one D-WSL at 5000x magnification with a 20.0 kV electron beam energy.

### Statistical analysis

Descriptive statistics for WSL depths were calculated according to pH cycle protocol and imaging modality. Enamel thickness and percent demineralized enamel were also calculated. The Pearson correlation between µ-CT and US was evaluated and interpreted according to Chan et al. [23] A linear mixed effects model was constructed to evaluate the reproducibility of US measurements on a subset of four samples measured three times in two separate US scans. US 1 and US 2 were considered two different modalities in this model, creating a total of 24 measurements. Additionally, ICC’s were calculated for the US 1 and US 2 measurements. The accuracy of US was analyzed with a linear model using the absolute difference between paired US and µ-CT measurements as the dependent variable. Signed bias in the US measurements was also evaluated with a linear mixed effect model and post-hoc contrasts using estimated marginal means from the model. The mixed effect model included a random intercept. Both models were assessed for normality of residuals by Q-Q plot and for homoscedasticity. Given the sample size, the observed deviations from normality were considered insufficient to justify transformation and preserve interpretability of estimates. Bland-Altman plots for both accuracy and signed bias were constructed. Ten randomly selected samples (5 in each imaging group) were re-measured one week apart, and intra-rater reliability of WSL depth measurements by the investigator were assessed using intraclass correlation coefficient (ICC). All analyses used a significance level of 0.05 and were performed using RStudio (version 2024.12.0 + 467).

## Results

### Intra-rater reliability

The intra-rater reliability of the lesion depth measurements (with 95% CI’s) was 0.998 (0.991, 0.999) for µ-CT and 0.986 (0.947, 0.997) for US, indicating a high reproducibility in both groups.

### µ-CT and ultrasound analysis

1. Reproducibility of Ultrasound:

With the understanding of likely low power and hence a high chance of a type II error, the mixed effect linear model found no significant differences between the repeated measures obtained from two US scans of four samples. ICC’s were also calculated for each US scans and were in line with the mixed model. The results for the first and second scan respectively (along with 95% confidence intervals) were 0.996 (0.977, 1) and 0.969 (0.847, 0.998). The S-WSL depths measured by US ranged from 128 μm to 159 μm for the first sample and 116 μm to 159 μm for the second sample. The D-WSL depths ranged from 382 μm to 434 μm and 276 μm to 340 μm.

**Fig. 2:**
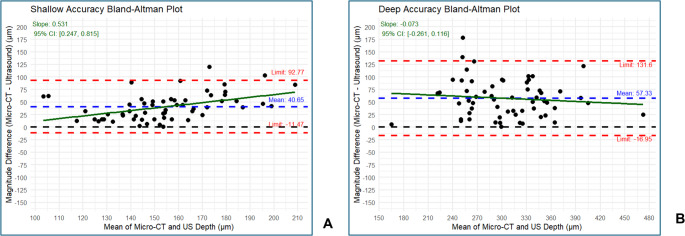
Bland-Altman plot showing the absolute difference of US and μ-CT for shallow (**a**) and deep (**b**) lesion accuracy values

2) Accuracy of Ultrasound:

Mean S-WSL and D-WSL depth measurements in both US and µ-CT are shown in Table 1 and Supplementary Fig. 3. Three samples were discarded because their US file was either lost or damaged (Fig. 2). The average depths for the S-WSL were 138.33 ± 17.84 μm and 169.22 ± 37.77 μm in µ-CT and US, respectively. Using the estimated marginal means from the linear model of absolute differences, or the accuracy, a mean difference of 40.65 μm (Table 2; Fig. 2a) was observed between µ-CT and US for the S-WSL. This difference was significantly greater than zero (*p* < 0.001).

**Table 1:** Descriptive statistics of 2-day (*n* = 58) and 4-day (*n* = 59) lesion depths (µm) in µ-CT and US

		WSL Depth (µm)				
pH Cycle Length	Modality	Range	Median	IQR	Mean	SD
2-Day	µ-CT	95.33–177.67	138.33	22.42	139.30	17.84
2-Day	US	72.67–251.00	169.00	47.67	169.22	37.77
4-Day	µ-CT	168–459.67	297.33	49.17	299.93	47.67
4-Day	US	162–321.33	321.33	121.67	309.28	75.66

*WSL* white spot lesion, *IQR* interquartile range, *SD* standard deviation

**Table 2:** Accuracy and bias of US in 2-day (*n* = 58) and 4-day (*n* = 59) lesion depth measurements

Measurement	pH Cycle Length	Mean (µm)	95% CI	*p*-Value*
Accuracy	2-Day	40.65	(32.12, 49.18)	< 0.001
	4-Day	57.33	(48.87, 65.78)	< 0.001
Bias	**pH Cycle Length**	**Estimate (µ-CT-US)**	**95% CI**	p-Value**
	2-day	−29.91	(−44.39, −15.44)	< 0.001
	4-day	−9.35	(−23.70, 5.00)	0.199

*CI* confidence interval

*Estimated marginal means from linear model of absolute differences (accuracy) between US and µ-CT

**Estimated marginal means contrasts (µ-CT – US) from linear mixed effects model of depth measurements

Regarding the D-WSL, the average depths were 299.93 ± 47.67 μm and 309.28 ± 75.66 μm in µ-CT and US, respectively. The absolute mean differences in depth measurements for the D-WSL were also significantly different from zero at 57.33 μm (*p* < 0.001; Table 2; Fig. 2b).

3) Signed Bias of Ultrasound

**Fig. 3:**
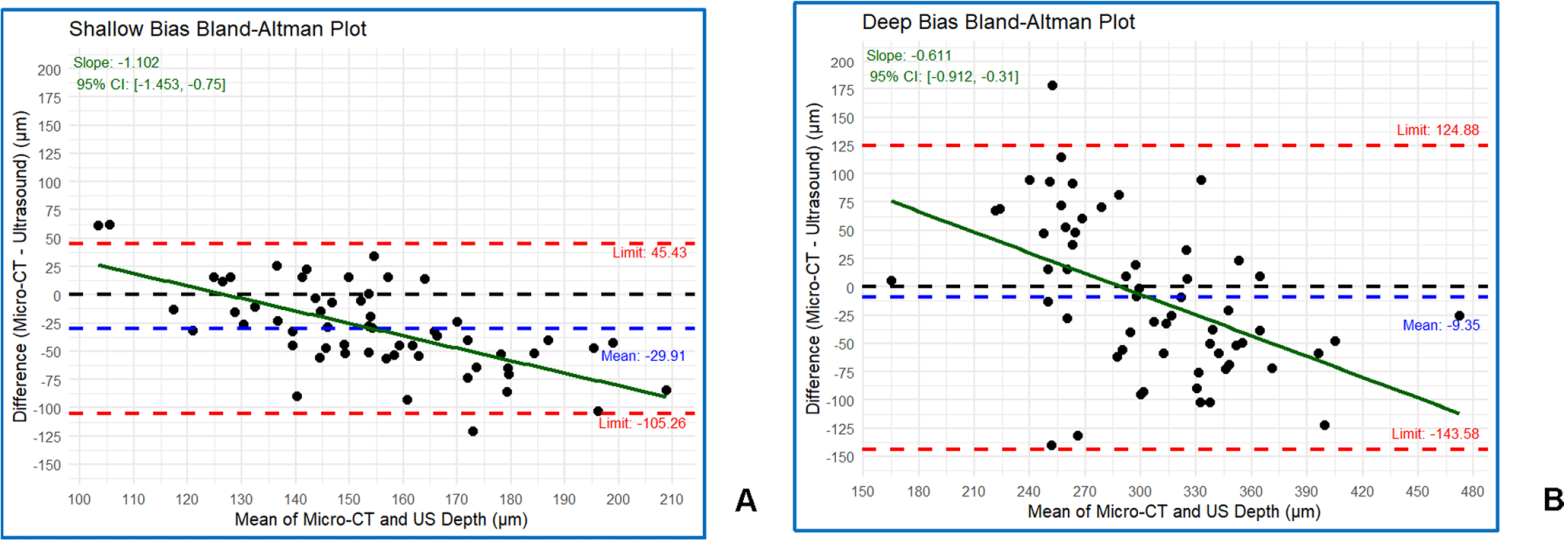
Bland-Altman plot showing the difference of US and μ-CT for shallow (**a**) and deep (**b**) lesion bias values

Regarding signed bias, the S-WSL measured on average 29.91 μm deeper in US than in µ-CT (*p* < 0.001; Table 2; Fig. 3a). Signed bias in the D-WSL was less than the S-WSL, with US showing an average depth of 9.35 μm greater than µ-CT (*p* = 0.199; Table 2; Fig. 3b).

**Fig. 4:**
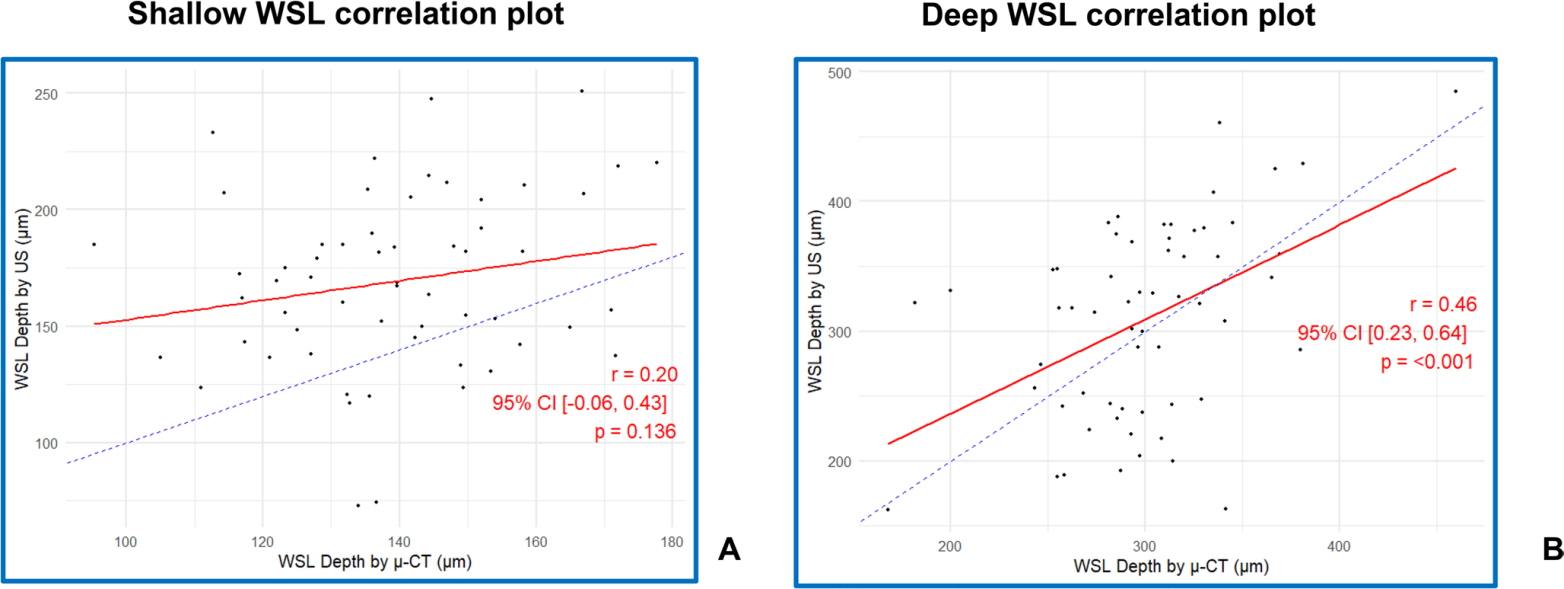
Correlation between US and µ-CT depth measurements in shallow (**a**) and deep (**b**) lesions (bisector line in blue)

While the average depths in the µ-CT and US were more closely aligned in the D-WSL (299.93 μm and 309.28 μm, respectively) compared to the S-WSL (139.3 μm and 169.22 μm, respectively), there was a greater variability in the D-WSL (Table 1). Furthermore, the divergence between the absolute differences was significant between the S-WSL and D-WSL (average difference = 16.70 μm, *p* = 0.007). There was a poor non-significant positive correlation in the S-WSL measurements between the two modalities (*r* = 0.20, 95% CI [−0.06, 0.43], *p* = 0.136) and a fair positive correlation in the D-WSL measurements (*r* = 0.46, 95% CI [0.23, 0.64], *p* < 0.001) (Fig. 4a-b).

The average enamel thickness in the S-WSL and D-WSL groups were 720.97 ± 203.68 μm and 803.28 ± 210 μm, respectively (Table 3). The average percentage of enamel demineralized was 22.60 ± 14.11% in the S-WSL and 40.70 ± 15.63% in the D-WSL.

**Table 3:** Enamel thickness and percent demineralized enamel of 2-day (*n* = 58) and 4-day (*n* = 59) lesion in µ-CT

Measurement	pH Cycle Length	Range	Median	IQR	Mean	SD
Enamel Thickness (µm)	2-day	136.33–1040.00	729.00	309.58	720.97	203.68
	4-day	255–1483.67	793.67	265.00	803.28	210.00
% enamel demineralization	2-day	10.91–100.00	19.09	8.90	22.56	14.11
	4-day	16.93–100.00	36.72	12.72	40.72	15.63

*IQR* interquartile range, *SD* standard deviation

**Fig. 5:**
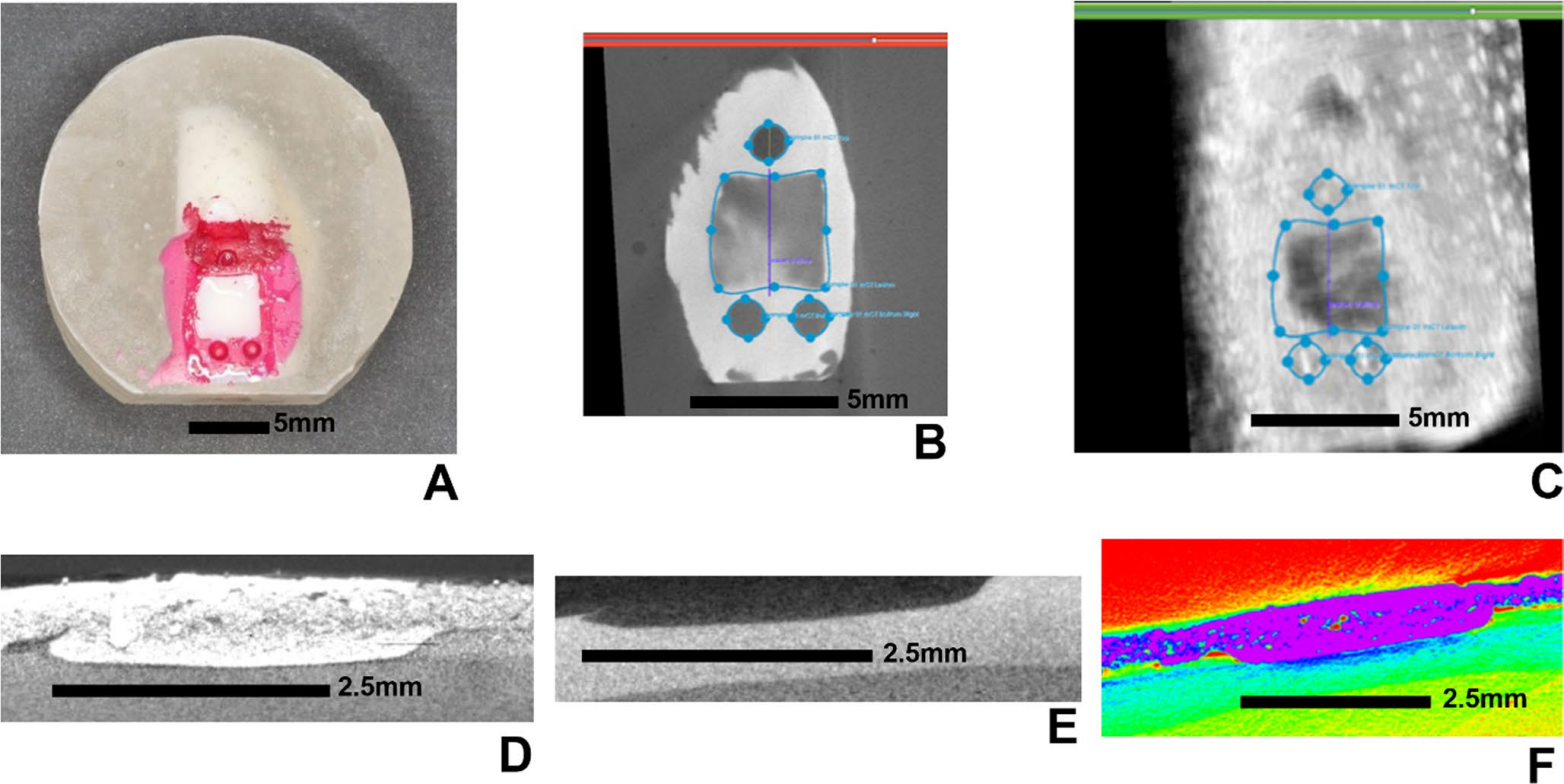
3D superposition of one sample’s US and µ-CT images. Image of the sample (**a**), outline of the lesion in µ-CT (**b**), µ-CT lesion outline superposed on US image of lesion (**c**), verification of WSL demineralization with barium sulfate (**d**), same sample without barium sulfate (**e**), and image a with the ImageJ spectrum lookup table to enhance visibility of the differences in density (**f**)

**Fig. 6:**
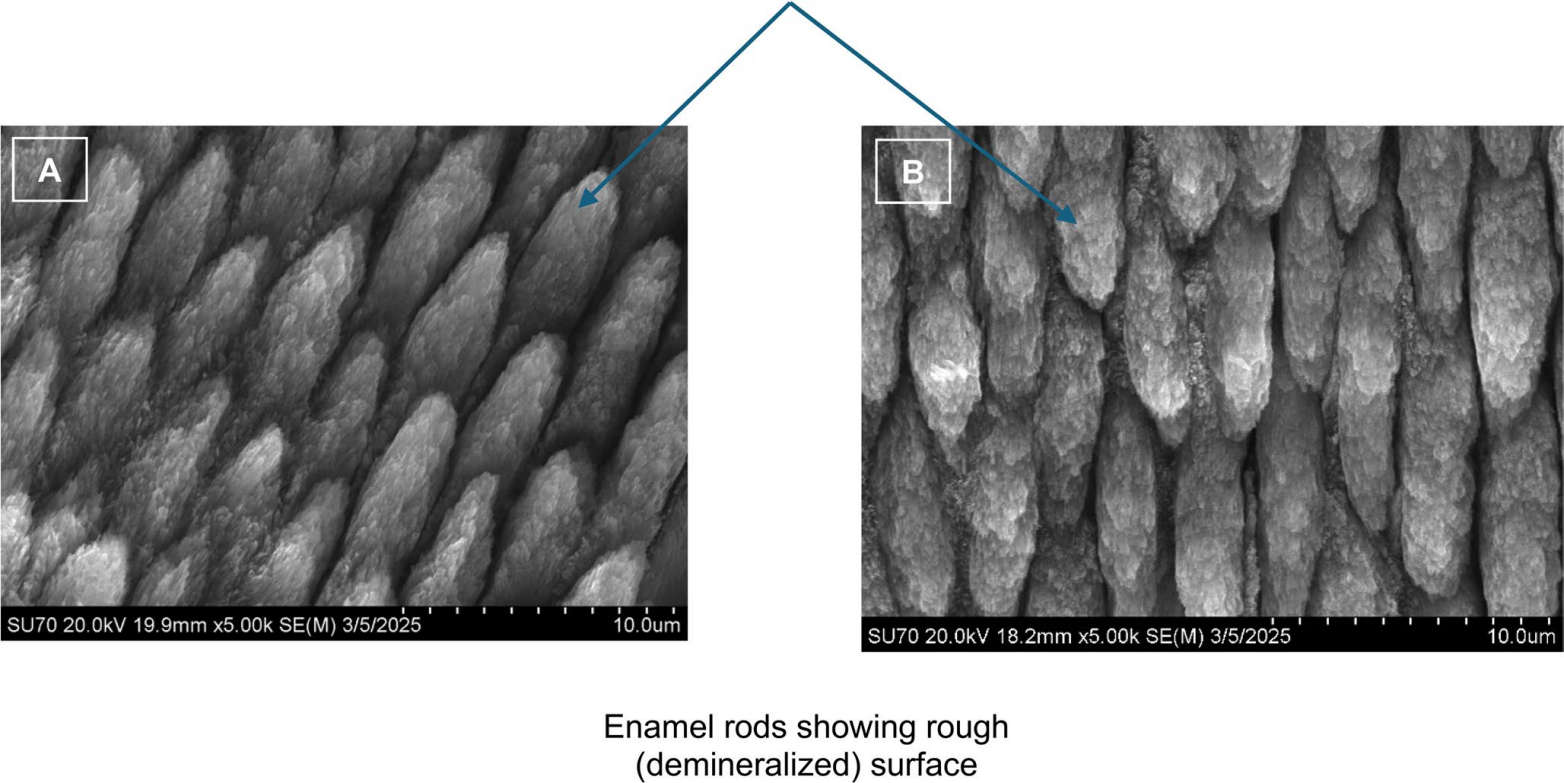
SEM images of a representative 2-day lesion (**a**) and a 4-day lesion (**b**) taken at 5000x magnification using carbon sputter coating and 20.0kV electron beam energy

Superposition of the US and µ-CT images of one WSL showed the surface area geometry and sagittal geometry was comparable (Fig. 5a-c). Results of the barium sulfate analysis showed that the barium sulfate and petroleum jelly mixture was able to penetrate the lesion, suggesting a high amount of demineralization (Fig. 5d-f). Analysis of the SEM radiographs revealed demineralized enamel rods at the WSL surfaces. The microscopic appearance of the demineralization was similar in the S-WSL and D-WSL and is representative of surface demineralization (Fig. 6).

## Discussion

This in vitro study aimed to validate US as a method for measuring the depth of WSLs by comparing the accuracy and bias of US and µ-CT in measuring WSL depth on bovine teeth. The study used the Zonare ZS3 US system for assessment of WSLs. The L30-8 high-frequency (24 MHz) transducer is small enough to be used intraorally, and previous studies have found that with the use of protective barriers and proper disinfection techniques, this transducer can be used among different patients while still creating high-quality output [15]. It is a linear array composed of 128 piezoelectric elements, a configuration that is useful for imaging shallow structures without the need for high lateral resolution [17]. A HFUS system was chosen to optimize image resolution, which is critical for this study. Although HFUS systems (> 15 MHz) offer reduced penetration depth compared to lower frequency systems, this limitation is irrelevant in WSL imaging, as lesions are shallow and located near the surface [11, 18]. The ZS3 system has been used in multiple dental studies [19–22].

This study aimed to create WSLs of varying depths to evaluate diagnostic accuracy across lesion severities, as treatment approaches may differ depending on lesion depth. Lesions that are non-cavitated and are confined to the outer two thirds of the enamel correlate to WSLs and an ICDAS I or II as outlined by Ekstrand, Ricketts and Kidd [23]. Roberts et al. state that intra-enamel caries is not visible as a WSL until the subsurface zone of demineralization reaches 400 μm, when the backscattering of light within the lesion is enough to visually detect it after 5 s of air drying [24]. The lesions created in this study, as measured by µ-CT, ranged from 95.33 to 459.67 μm. The average percent demineralized enamel ranged from 22.6% in S-WSL to 40.7% in D-WSL. All but five lesions were confined to the outer two thirds of the enamel. It is important to note that the tooth surfaces were polished prior to WSL creation to establish a consistent reference for the outer surface, resulting in some initial enamel loss. This initial enamel loss was difficult to quantify, due to inherent biological variability in the bovine teeth used and the force/time method of material removal employed during polishing. However, among more than one hundred specimens, only five exhibited WSL penetration beyond the enamel, leading the authors to conclude that the experimental setup did not compromise the estimated percentage of demineralization.

This study investigated the accuracy of US in measuring WSL depth as compared to µ-CT. The study found that US is less accurate than µ-CT because the absolute differences between the means were significantly different from zero in both S-WSL and D-WSL. Previous studies on the use of US to measure the depth of WSLs have claimed evaluation of the accuracy of the modality [5]. However, they were either reporting values related to presence or absence of a lesion such as sensitivity and specificity [6] or were in fact reporting bias and not taking into account over or under-estimation of depth measurements in their analysis [12]. By not looking at absolute values of mean differences, the results include both negative and positive variations of US measurements from the gold standard measurements. Therefore, positive and negative values can cancel out, making the average differences skew toward zero. To truly assess whether the average differences are significantly different, absolute values must be used.

This study also found a weak, statistically non-significant positive correlation between the S-WSL measurements in the two modalities (*r* = 0.20, 95% CI [−0.06, 0.43], *p* = 0.136) and a fair positive correlation in the D-WSL measurements (*r* = 0.46, 95% CI [0.23, 0.64], *p* < 0.001) [25]. This partially supports findings from Caliskan Yanikoglu et al. who compared depth measurements of WSLs between US and light microscopy and found that there was a weak, negative correlation between the two groups (*r* = −0.25, *p* = 0.279) [5]. Current study findings contradict results from Kim et al. who found that the differences between US and µ-CT depth measurements was not significant at *p* = 0.075 [11]. However, these results were based on a limited dataset of 20 images without repeated measurements. Additionally, the measurements were conducted using a biomicroscopic US system, which is not applicable for in vivo use. None of these studies identified on the topic considered the absolute value of the mean differences, which is a true estimation of accuracy. One possible explanation for the stronger correlation between µ-CT and US measurements in deeper WSL, compared to shallower lesions, is the increased surface porosity of the lesion, which can alter the US reflections received at the surface [11]. Additionally, to accurately measure the depth of a defect, the speed of sound of the material the waves are propagating through must be known. Previous studies have found that demineralized enamel has a speed of sound between dentin (3,800 m per second [m/s]) and pulp (1,570 m/s), with the minimum mean difference of depth between US and µ-CT being 77.78 μm when the pulpal sound speed was used [11]. The US system used in the present study operated at a soft-tissue speed of sound of 1,540 m/s, and no correction factor was applied to the depth measurements. The literature does not provide conclusive values for the speed of sound in WSLs; moreover, the speed of sound varies with lesion density, indicating that no single value is appropriate for all lesions [7].

The second aim of this study was to assess bias of US depth measurements compared to µ-CT. There was a greater difference in means between US and µ-CT depth measurements in S-WSL than in D-WSL with a significant difference noted in the S-WSL. This indicates that US tends to overestimate WSL depths in S-WSL, which may lead to overtreatment of demineralization. Such overtreatment could lead to unnecessary irreversible restorative interventions and removal of sound enamel. This result partially aligns with Yanikoglu et al., who assessed the diagnostic performance of US against microscopy as the gold standard [12]. They found the highest mean difference between US and microscopic measurements was in the shallowest lesions (ICDAS I, 0.58 ± 0.09 mm mean difference) and the lowest mean difference was in the deepest lesions (ICDAS III, 0.45 ± 0.16 mm). In other words, as lesions got deeper, mean differences became smaller. However, it must be considered that they reported mean differences while the present study reported absolute mean differences. Also, only mean differences in lesion depth were reported, leaving it unclear which modality measured deeper. Future studies should incorporate a wider range of lesion depths to better asses depth-related bias in US measurements. If a consistent bias is identified, correction factors can be applied to adjust measured lesion depths using calibration or regression-based methods. These corrections could enhance the comparability between modalities and minimize the risk of misclassification.

Overall, the results of the present study reject the null hypothesis that there is no significant difference between US and µ-CT in diagnosing the depth of demineralization in enamel. There are some limitations to US as highlighted in the current study. Methodological limitations include the use of bovine teeth for WSL depth measurements, which may limit the direct transferability of the findings to clinical conditions in human dentition. Additionally, the carious lesions were artificially induced under controlled laboratory conditions; therefore, they may not accurately replicate the complexity, progression, and etiological factors of naturally occurring caries in vivo, which are influenced by multifactorial biological and behavioral risk factors. To establish a baseline for lesion initiation, flat surfaces were polished on each sample tooth. This polishing may have altered the initial enamel thickness and the calculated percentage of demineralization, as enamel nanostructure varies across its depth. Additionally, the investigator was not blinded to the samples as they were measured, although care was taken to measure the US and µ-CT images of each lesion at separate times. Future studies could enhance blinding by using independent measurers or randomized, masked image files.

In relation to US, the parameters described in this study (e.g., bandwidth, gain, pulse length, and environmental conditions) should be interpreted as controlled experimental variables rather than clinically validated operating conditions. These factors, along with additional real-world variables, will need to be systematically evaluated and optimized in future in vivo and clinical studies before any claims regarding intraoral applicability can be made. Additionally, the cost and footprint of US equipment must be considered, along with the need for supplemental materials such as sterile gel to serve as a coupling agent, and training and calibration [5, 12, 15]. While US is objective, non-invasive, and capable of directly measuring lesion depth intra-orally rather than merely providing a signal that correlates with it, future studies should focus on optimizing US frequencies and applying advanced frequency filtering techniques to enhance the isolation of US echoes, thereby improving diagnostic accuracy in both shallow and deep WSLs. Additionally, studies may focus on determining WSL depths adjacent to orthodontic brackets and evaluating remineralization strategies as evidence from fluoride-release studies demonstrate that mineral changes around brackets are spatially heterogeneous and can influence imaging contrast [26].

## Conclusions

The absolute differences between US and µ-CT are significantly different from zero, suggesting US is not accurate when compared to the gold standard. Across all lesion severities, US on average, measured deeper than µ-CT, with the 2-day lesions measuring significantly deeper in US than in µ-CT. In contrast to µ-CT, high frequency US demonstrates potential for clinical use. However, extensive optimization and calibration are necessary before its reliable application in the clinical assessment of WSL depth.

## Supplementary Information

Below is the link to the electronic supplementary material.

Supplementary Material 1 (DOCX 134 KB)

## Data Availability

The micro-CT DICOM files and Ultrasound TIFF stacks used and/or analyzed during the current study are available from the corresponding author on reasonable request.
