# Supplementary material for: Accuracy and bias of high-frequency ultrasonography in measuring white spot lesion depth: an in-vitro comparison with micro-CT
Source: Clin Oral Investig. 2026 Jun 3;30(6):267. doi: 10.1007/s00784-026-06956-y (PMC13234065; doi:10.1007/s00784-026-06956-y)

**Figure 1- Suppl:** Study procedures

**Figure 2 – Suppl:** Study CONSORT diagram

**Figure 3 – Suppl:** S-WSL (a) and D-WSL (b) per-sample paired plots.


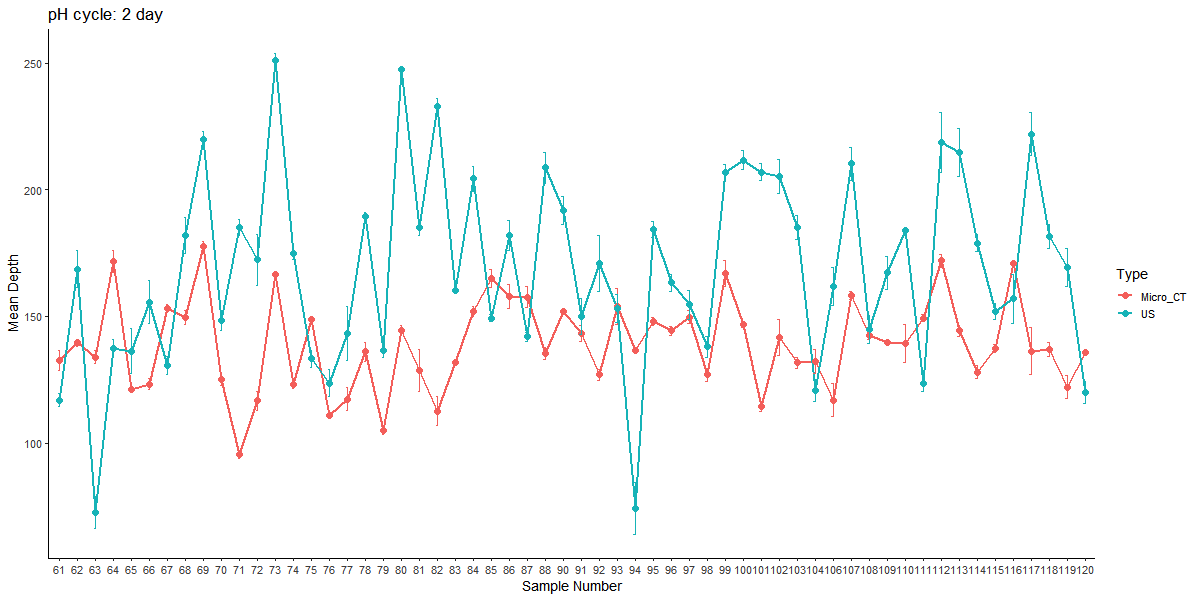


(a)


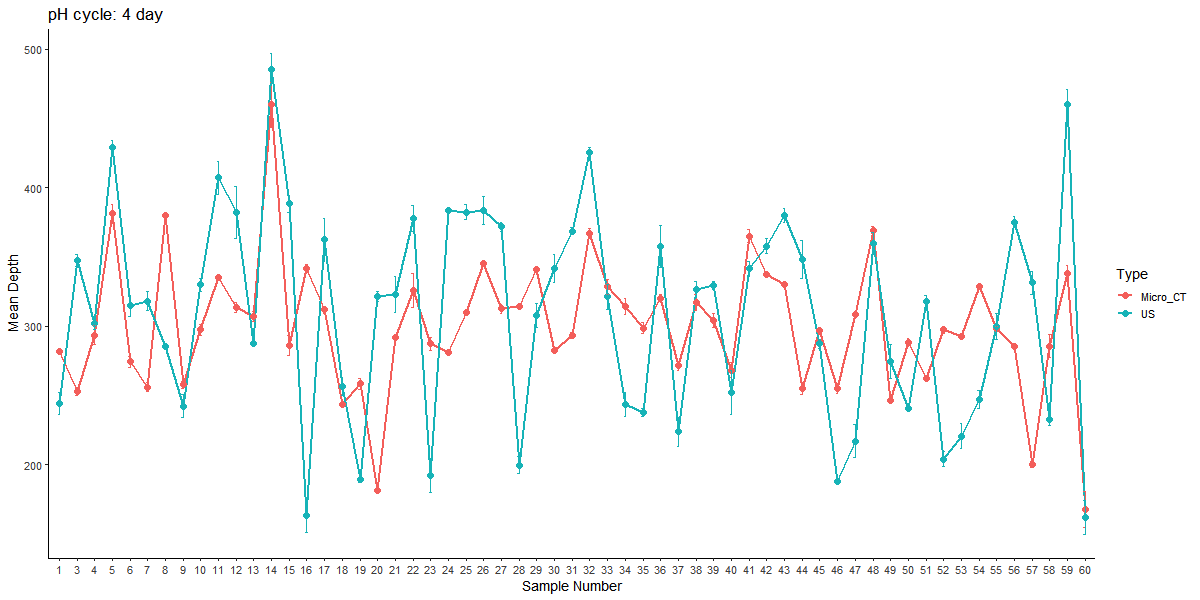

Supplement: Supplementary file 1 — Supplementary Material 1 (DOCX 134 KB) [file 784_2026_6956_MOESM1_ESM.docx]
